# Detection of chikungunya virus in the Southern region, Saudi Arabia

**DOI:** 10.1186/s12985-021-01660-7

**Published:** 2021-09-20

**Authors:** Abdulrahim R. Hakami, Abdullah A. Alshamrani, Mohamad Alqahtani, Yasser Alraey, Razan A. Alhefzi, Sultan Alasmari, Mohamed Makkawi, Gasim Dobie, Mushtaq Mir, Mohamed Alshahrani, Ayed Dera, Mohammed Alfaifi, Mesfer Al Shahrani, Ahmad Matari, Ali Essa Asiry

**Affiliations:** 1 Department of Clinical Laboratory Sciences, College of Applied Medical Sciences, King Khalid University, Abha, 61481 Saudi Arabia; 2 Department of Medical Laboratory Technology, Jazan University, Jazan, Saudi Arabia; 3 Department of Hematology and Blood Bank, Baish General Hospital, Jazan, Saudi Arabia; 4 Department of Serology, Asir Central Hospital, Abha, Saudi Arabia

**Keywords:** Chikungunya, Hemorrhagic fever, Mosquitoes, Thrombocytopenia

## Abstract

**Background and aim:**

Despite the fact that the chikungunya viral infection is a neglected disease, complications such as hemorrhagic fever, arthritis, and lymphopenia remain a health concern. The aim of this study was to determine the prevalence of the chikungunya virus in the Southern Region, Saudi Arabia. Enzyme immunoassay and polymerase chain reaction have been compared between samples.

**Materials and methods:**

Forty samples from two southern hospitals in Saudi Arabia were collected between December 2019 and February 2020 and screened for chikungunya virus IgG antibodies and for viral RNA. Selection criteria were based on hematological parameters and rheumatological profiles such as rheumatoid factor, c-reactive protein, anti-nuclear antibody, and anti-cyclic citrullinated peptide (anti-CCP) of out-patients.

**Results:**

One confirmed case of chikungunya virus was detected using the ELISA test. However, no viral RNA was detected in any of the samples. This suggests that the virus is cleared rapidly in patients.

**Conclusion:**

Chikungunya is a neglected viral disease in Saudi Arabia. Future work should focus on detailed investigation of this viral infection and its vectors.

## Introduction

Arboviruses are arthropod-borne viruses that can be transmitted from ticks and mosquitoes to humans and animals. Chikungunya virus belongs to the *Togaviridae* family and alphavirus genus that is transmitted by mosquitoes, and is considered a neglected tropical disease [1]. The Jazan Region, Saudi Arabia, lies within the tropical zone, and is known by its hot climate. It is dominated by mosquitoes that can transmit hemorrhagic viruses such as dengue. Rift Valley fever (RVF) virus is another arbovirus that emerged in the Jazan Region for the first time in 2000 [2].

Arboviruses, which include the families *Flaviviridae* and *Togaviridae*, cause hemorrhagic fever which is known as “bone-breaker fever” [3]. In Saudi Arabia, dengue cases between 2013 and 2015 have been reported mainly in Makkah and Jeddah, while very few cases were reported in the Jazan and Asir regions [4]. Moreover, the chikungunya virus was reported in Jeddah for the first time in 2011 following a positive quantitative real-time polymerase chain reaction (qRT-PCR) result for a 55-year-old women who suffered from severe arthralgia [5]. IgM and IgG antibodies against the virus were detected. The chikungunya virus is characterized by rapid onset of fever, short viremia, rash, arthralgia, skin bleeding, hemorrhagic dyscrasia, joint swelling, elevated c-reactive protein, leukopenia, and normal or low platelet count [6]. Other reported manifestations include seizure, jaundice, gastrointestinal bleeding, pulmonary hemorrhage, intracranial hemorrhage, conjunctival bleeding, cyanosis, and even death in neonates [7–11].

Mosquito diversity and abundance vary between the Asir and Jazan regions due to the huge climatic and geographical variation between the two regions [12, 13]. In general, the *Aedes* species have not been reported at the highlands of the Asir region. Nevertheless, the endemicity of dengue virus has been confirmed only in the Tihamah part of the Asir region (Tihāmat Asīr) which is similar to the Jazan region in terms of climate conditions [14, 15], which suggests the prevalence of *Aedes* species in this area. The most significant human viral diseases including dengue, chikungunya, and zika are mainly transmitted globally by *Aedes aegypti* and *Aedes albopictus*. Therefore, controlling the *Aedes* species using insecticide and providing risk maps with vector populations will help to reduce the arboviruses transmission [16]. To the contrary, the threat of insecticide resistance to multiple insecticides (e.g. pyrethroids and organophosphates) still exists, and, therefore, looking for an alternative control measurements is highly recommended [4, 17]. The objective of this study was to determine whether the chikungunya virus is circulating in two different Saudi Arabian southern regions by comparing the results of enzyme-linked immunosorbent assay (ELISA) and PCR.

## Materials and methods

### Sample collection

**Fig. 1:**
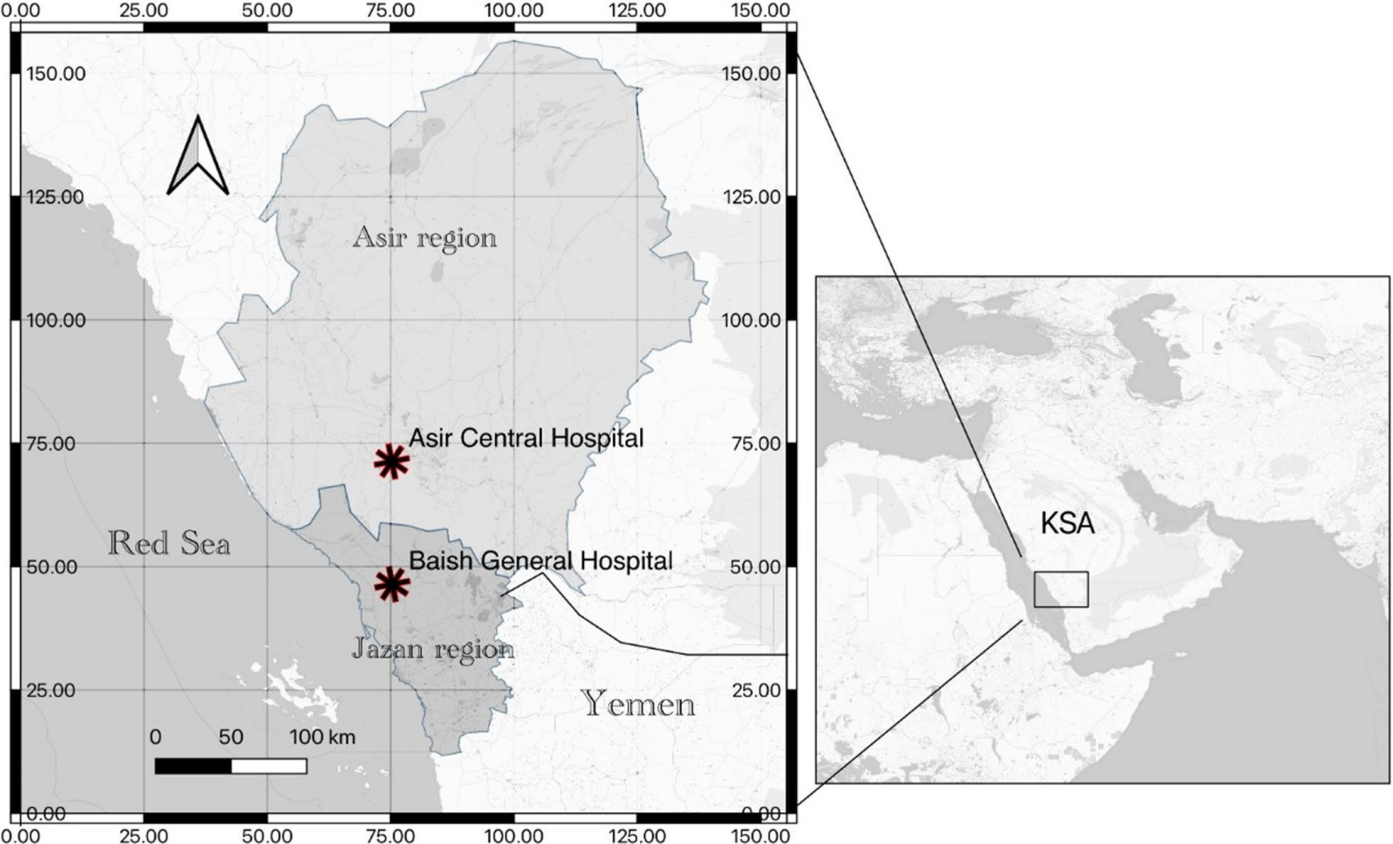
A map of the locations from which samples were collected. Image created using QGIS Geographic Information System; QGIS.org

To increase the chance for detection of an arbovirus that has not been extensively studied, inclusion criteria should focus on proper sample selection. The most common clinical presentations of arboviruses including the chikungunya virus are joint pain and bleeding. Abnormal platelet and white blood cells (WBCs) are other important laboratory profiles. Serum samples from arthritis patients and patients with hemorrhagic fever were collected from different altitudes: 30 serum samples from Asir Central Hospital and 10 patients from Baish General Hospital in the Jazan Region (Fig. 1), where *Aedes aegypti* mosquitoes thrive. The collection was conducted in the winter season between December 2019 and February 2020 where cases of hemorrhagic fever dominate. At Asir Central Hospital, 30 samples were selected after they had been sent to the laboratory based on the physicians’ requests for rheumatological profiles (rheumatoid factor, c-reactive protein, anti-nuclear antibody, and anti-cyclic citrullinated peptide (anti-CCP) of out-patients). Those patients were clinically diagnosed with rheumatoid arthritis. At Baish General Hospital, samples from patients with nasal bleedings who were admitted to the hospital were selected.

### Detection of anti-chikungunya virus IgG

Anti-chikungunya virus IgG class antibodies were qualitatively measured using enzyme immunoassay with the sandwich type based on avidin–biotin binding. Anti-human IgG antibodies were pre-coated on the solid phase of 96-well microplate to cross-react with human antibodies in the serum. Following the addition of the controls and the 1:100-dilted serum samples, wells were washed. The chikungunya virus antigen (abcam, ab177835) was then added. After one hour of incubation time, chikungunya virus biotinylated antibodies were added, incubated, and washed, followed by the addition of streptavidin peroxidase conjugate. Tetramethylbenzidine (TMB) is catalyzed by the peroxidase enzyme. Absorbance was measured using a spectrophotometer at 450 nm wavelength using FLUOstar Omega (BMG LABTECH GmbH, Germany), and the color is directly proportional to the captured anti-chikungunya virus IgG antibody. Data and figures were presented using GraphPad Software, version 8, (San Diego, CA, USA).

### Extraction of viral RNA

Viral nucleic acid was extracted using ABIOpure Viral, version 2.0, DNA/RNA Extraction kit, #M561VT50, AllianceBio, USA. This uses advanced silica-binding technology to purify viral RNA that binds to the silica membrane, and finally the eluate was collected in a clean tube. The protocol was performed using 10 μl proteinase K, 200 μl sample, 7 μl carrier RNA to enhance binding of viral nucleic acid to the spin column membrane, 200–400 μl of the intended buffer, and 20 μl nuclease-free water.

### Reverse transcription PCR

**Fig. 2:**
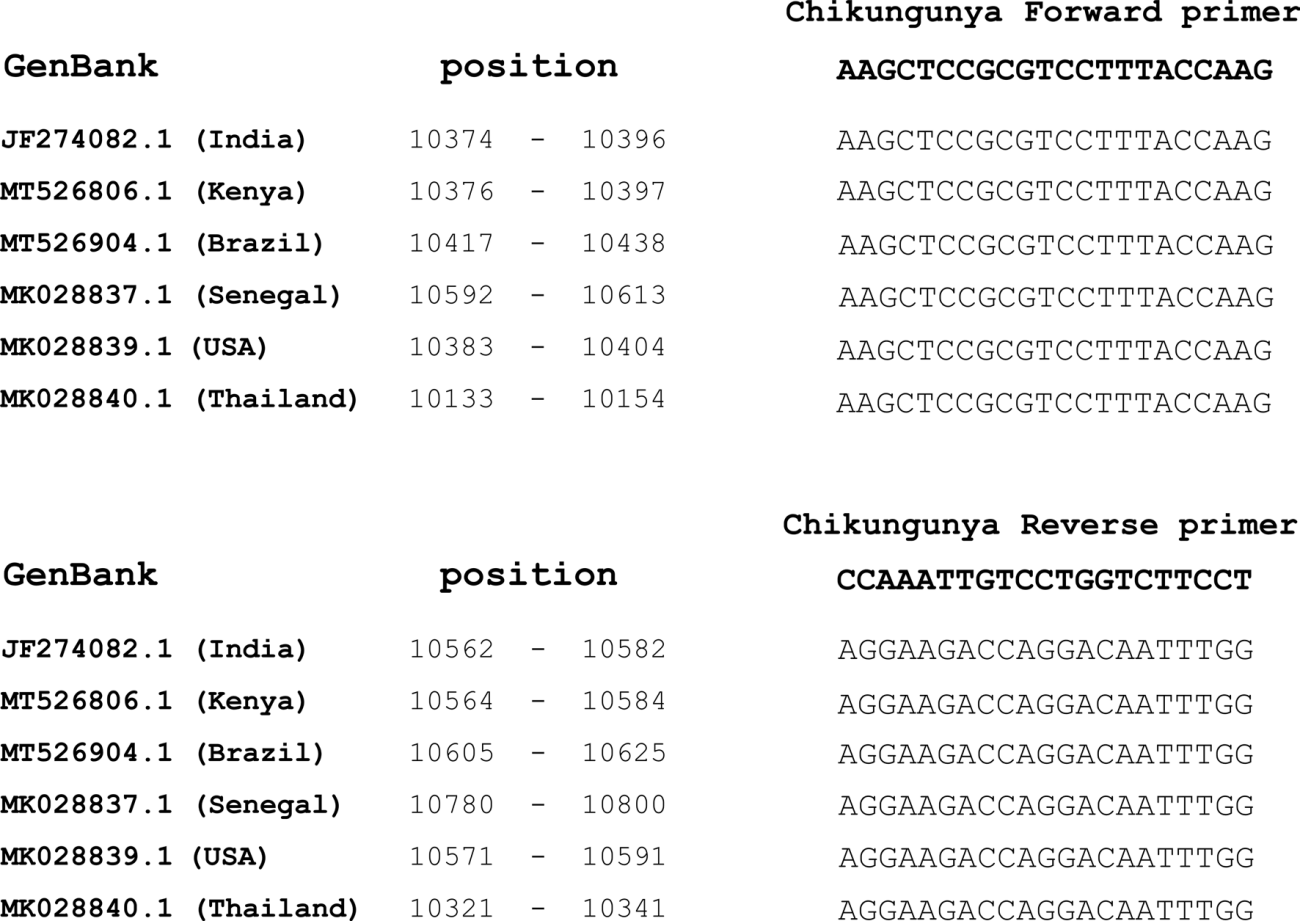
Mapping of primer domains of different chikungunya virus isolates

The following materials were obtained from meridian Bioscience™, or otherwise stated. Reverse transcription polymerase chain reaction (RT-PCR) was used to detect the genome of chikungunya virus in blood samples. The forward primer AAGCTCCGCGTCCTTTACCAAG and the reverse primer CCAAATTGTCCTGGTCTTCCT [18] were synthesized by Eurofins Genomics, Germany (Table 1). The primer domains are conserved among chikungunya isolates that target the E gene (Fig. 2). The cDNA synthesis was performed according to SensiFAST™ cDNA Synthesis Kit protocol. The master mix was prepared on ice, and centrifuged briefly. One microgram of the viral RNA was used with 4 μl of buffer and 1 μl of reverse transcriptase enzyme. The reaction volume was topped up to 20 μl with nuclease-free water, and mixed gently by pipetting. The thermal cycler was set up at 25 °C for 10 min (primer annealing), 42 °C for 15 min (reverse transcription), 8 °C for 5 min (inactivation), and then chilled on ice. To perform the PCR, the master mix and the cycling parameters were conducted according to the protocol of MyTaq™ Mix, meridian Bioscience™, United Kingdom (Table 1). RT-PCR was performed using BIO-RAD T100™ Thermal Cycler.

**Table 1:** List of chikungunya primers and PCR cycling parameters

Primers	Sequence	Size (bp)
Forward CHIK 1	AAGCTCCGCGTCCTTTACCAAG	208
Reverse CHIK 1	CCAAATTGTCCTGGTCTTCCT	

Cycling parameters			
Step	Cycles	Temperature	Time
Initial Denaturation	1	95	1 min
Denaturation	35	95	15 s
Annealing		55	15 s
Extension		72	10 s
Final extension	1	72	10 s

## Results

### A positive case of the chikungunya virus

**Fig. 3:**
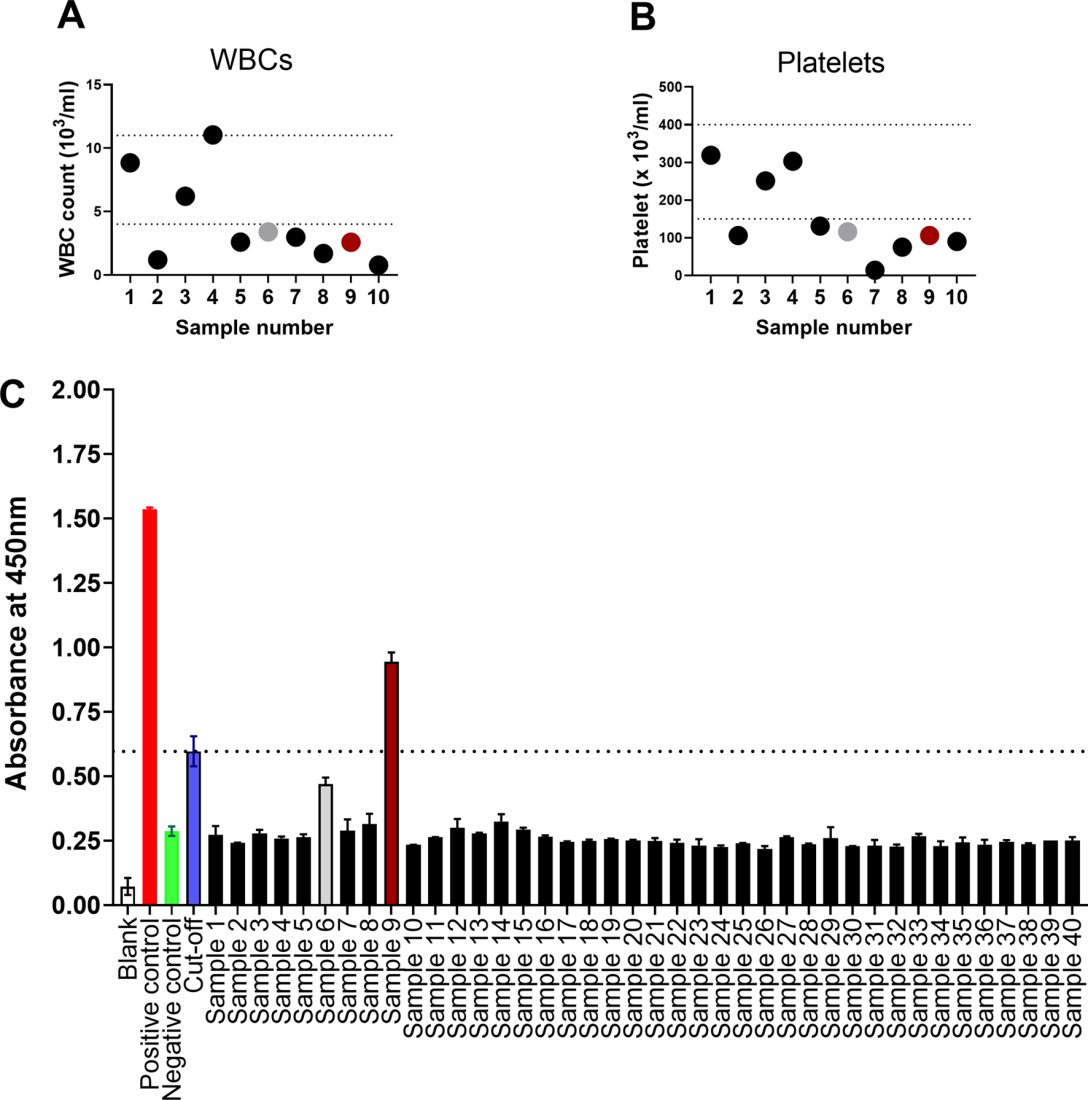
CBC data and anti-chikungunya IgG results. Levels of WBCs (**A**) and platelets (**B**) of the first group of patients that were collected from Baish General Hospital (Samples 1 to 10) in which a confirmed chikungunya case was detected (Sample 9; scarlet) and an equivocal result (Sample 6; grey). (**C**) Results of anti-chikungunya virus IgG for all patients including arthritis patients that were collected from Asir Central Hospital (Samples 11 to 40). The dotted two lines (**A** and **B**) indicate the normal range. The dotted line (**C**) indicates the cut-off value. Error bars represent ± SD of the mean

Rheumatological and hematological parameters of patients were considered. Patients with abnormal CBC and those who had been provisionally diagnosed with rheumatoid arthritis to whom samples were received at the hospital laboratory were the target of this study. Two groups of patients’ samples were tested for the presence of anti-chikungunya virus and viral RNA detection. They were from Baish General Hospital (low altitude) in which ten samples were collected with abnormal CBC, and the second group was from Asir General Hospital (high altitude) who suffered from arthritis, in which 30 samples were collected. Only one sample was positive for the anti-chikungunya IgG antibodies, and one equivocal sample of duplicate tests. These two samples were from the first (low-altitude) group in which the *Aedes* mosquito thrives. They both showed abnormal low WBC and platelet counts (Fig. 3; Samples 6 and 9).

### Negative PCR tests suggest rapid clearance of chikungunya virus

All samples were screened to detect chikungunya virus RNA following the viral extraction protocol, cDNA synthesis, and PCR. Gel electrophoresis was then prepared at a concentration of 2% agarose gel using UltraPure™ Agarose, Invitrogen, 1X TAE buffer, and ethidium bromide (Sigma). Samples were loaded with DNA loading buffer and with HyperLadder 100 bp (catalog number BIO-33056). No bands were observed for any samples. This suggests that the viremic state of the chikungunya patient is very short.

## Discussion

During the winter season, patients were provisionally diagnosed with dengue fever virus, but no detection kits specific for arboviruses were available at the hospitals’ laboratories that time. Patients with low WBC and low platelet count were selected from Baish General Hospital. Another cohort from Asir Central Hospital was included in the screening, using samples from patients who suffered from arthralgia. Another criterion for inclusion was the positivity of anti-CCP and rheumatoid factor (RF). These markers aided in the diagnosis of rheumatoid arthritis patients [19].

While the dengue virus caused severe morbidity and mortality in Saudi Arabia, the Southern Region is at risk of other arboviruses due to outbreaks in neighboring Yemen [20–24]. Dengue is not the only viral infection that is endemic in Saudi Arabia, but as chikungunya virus was also discovered. The Jazan Region in Saudi Arabia is a suitable habitat for *Aedes* mosquito breeding; the vector of the dengue and chikungunya virus. Although hemorrhage is considered a rare complication in chikungunya infection, the upsurge of hemorrhagic fever cases in Jazan during the breeding period of mosquitoes led us to investigate the prevalence of chikungunya infections in arthritic and hemorrhagic fever patients.

Patients’ sera were screened for the presence of specific IgG antibodies to chikungunya virus. Platelet and WBCs count of seven patients from the first group were below the normal range. However, only one patient was found to be positive for anti-chikungunya IgG antibodies. In addition, sera were tested for the presence of the viral E gene. However, results were all negative using RT-PCR. We believe that the virus was rapidly cleared by the host immune system. Having negative results for PCR suggests that the virus is characterized by a short viremic state. Infected patients clear the virus after one week of chikungunya infection [25].

Finally, it is important to note the limitations. Selecting specific samples that fit the inclusion criteria at the peak of infection from different locations would be challenging. Viral hemorrhagic cases are soared only in certain periods throughout the year. This drawback limited our ability to satisfactorily increase the sample size. Because the likelihood of detecting the chikungunya virus was low, and because PCR results were negative, the control sample size was moderate. Following initial chikungunya infection, the virus is shed after approximately one week. Therefore, the detection probability of viral nucleic acid would be restricted. The study is limited by the small sample size; however, samples from two different hospitals were collected. Patients might have arrived at the hospital after a week of the infection, and therefore, molecular detection of chikungunya was challenging and not possible, at least during our study. It was not possible to test IgM. Instead, molecular detection of the viral RNA was attempted to detect the acute phase of the chikungunya virus. While the background noise of the ELISA test seems somewhat high, the cut-off value (COV) was set as a basis for the results interpretation. COV average (0.597 optical density) was higher than the negative control (0.287). Most samples were within the negative control range.

## Conclusion

Out of 40 samples, one confirmed case of chikungunya virus was found after detecting anti-chikungunya IgG antibodies, but PCR showed negative results in all tested samples. Detection methods for the chikungunya virus are not applied routinely, and the virus might be circulating in regions where mosquitoes are abundant. Arboviruses such as the chikungunya virus should be considered a priority with health implications. There have been reports of death due to bleeding. A call to conduct extensive studies on such neglected infections is indeed required. Chikungunya is a neglected viral infection, and future work should focus on investigating the endemicity of arboviruses within the vector by performing a field work collection of *Aedes* species within selected areas. To avoid a large impact of any conclusion drawn from the small samples, a large sample size should be collected for future investigations.

## Data Availability

All data generated or analyzed are presented in the manuscript. Accession numbers of chikungunya isolates are available at http://ncbi.nlm.nih.gov/nuccore/

## Abbreviations

Anti-CCP: Anti-cyclic citrullinated peptide
COV: Cut-off value
ELISA: Enzyme-linked immunosorbent assay
qRT-PCR: Quantitative real-time polymerase chain reaction
RT-PCR: Reverse transcription polymerase chain reaction
RVF: Rift valley fever
RF: Rheumatoid factor
TMB: Tetramethylbenzidine
WBC: White blood cell
